# Spatial pattern and environmental determinants of benthic diatom diversity in the middle and lower reaches of the Yellow River

**DOI:** 10.3389/fmicb.2025.1677888

**Published:** 2025-10-16

**Authors:** Qingyang Guo, Qin Zhu, Pengfei Peng, Qiuling Zhou, Yong Ding, Zhenchang Zhu

**Affiliations:** 1 Guangdong Basic Research Center of Excellence for Ecological Security and Green Development, Guangdong Provincial Key Laboratory of Water Quality Improvement and Ecological Restoration for Watersheds, Guangdong University of Technology, Guangzhou, China; 2 Southern Marine Science and Engineering Guangdong Laboratory (Guangzhou), Guangzhou, China; 3 Guangdong Provincial Observation and Research Station for Social-Natural Complex Ecosystems in Haizhu Wetlands, Guangzhou, China; 4 South China Sea Marine Survey Center, Ministry of Natural Resources, Guangzhou, China; 5 Central Cycleecological Technology Co., Ltd., Guangzhou, China

**Keywords:** Yellow River, benthic diatoms, biodiversity, water quality, land use

## Abstract

**Introduction:**

Benthic diatoms are critical bioindicators of freshwater ecosystem health due to their responsiveness to environmental shifts. However, previous studies have often overlooked the integrated effects of environmental, geographical, and anthropogenic factors on diatom diversity, particularly in sediment-laden and highly heterogeneous rivers like the Yellow River.

**Methods:**

This study examines the spatial patterns and environmental determinants of benthic diatom diversity in the middle and lower reaches of the Yellow River. Data were collected from 46 sampling sites, incorporating environmental, geographical, and anthropogenic factors. Key water quality parameters, including total nitrogen (TN), total phosphorus (TP), and turbidity, were analyzed. Structural equation modeling was employed to assess the mediating role of water quality in linking land use patterns and geographical gradients to diatom community structure.

**Results:**

Analysis identified TN, TP, and turbidity as primary drivers of diatom diversity. The middle reaches exhibited higher alpha diversity, associated with moderate nutrient levels and stable hydrological conditions. In contrast, the lower reaches, influenced by urbanization and nutrient enrichment, showed community homogenization and dominance by eutrophic taxa.

**Discussion:**

By investigating multi-scale drivers of diatom diversity in a heterogeneous and human-impacted river system, this study proposes a framework for analyzing biodiversity patterns. It provides insights for sustainable management of the Yellow River and comparable large river systems.

## 1 Introduction

Freshwater ecosystems rank among the most critical and vulnerable habitats globally (Sotnikova et al., 2024), contributing significantly to biodiversity maintenance, water cycle regulation, and ecosystem services such as water purification and nutrient cycling (Tang et al., 2024). Rivers, as integral components of these ecosystems, connect terrestrial and aquatic environments and support a wide range of biodiversity, including benthic diatoms (Hao et al., 2024; Yue et al., 2023). The Yellow River, China's second-largest river, holds substantial ecological and socio-economic importance (Xu et al., 2024). Stretching over 5,000 kilometers across nine provinces, it sustains more than 100 million people (Gao et al., 2024), supplying water for agriculture, industry, and domestic needs. Known for its heavy sediment load—earning it the nickname “the Mother River”—the Yellow River has shaped China's agricultural heartland for millennia. It is often referred to as “the cradle of Chinese civilization”, reflecting its historical significance (Zhang et al., 2024b). However, the ecological health of the Yellow River is under severe threat due to increasing anthropogenic pressures, such as urbanization, agriculture, and industrial activities, which degrade water quality and biodiversity (Li et al., 2024; Ren and Liu, 2024).

Benthic diatoms, a prominent group of microscopic algae, are established bioindicators of freshwater ecosystem health due to their rapid sensitivity to environmental changes (Baharuddin et al., 2024). Occupying the interface between water and sediment, benthic diatoms play critical roles in primary production, nutrient cycling, and the stabilization of sediments (Smeti et al., 2023). Their species composition and diversity are strongly influenced by water quality parameters such as nutrient levels, turbidity, and hydrological conditions, making them effective tools for assessing ecological integrity and monitoring anthropogenic impacts on aquatic systems (Tornés et al., 2022). Studies of diatoms have provided valuable insights into the effects of nutrient enrichment, eutrophication, and pollution in rivers, lakes, and reservoirs worldwide (Lu et al., 2022). For instance, research on large river systems such as the Rhine and Yangtze has shown that diatom communities are closely linked to nutrient gradients and sedimentation patterns, reflecting both natural and human-induced environmental changes (Lacroix et al., 2007; Liu et al., 2016). Additionally, diatom-based ecological assessments are widely used in water quality management due to their established relationships with specific environmental thresholds, such as the trophic state and nutrient levels (Desrosiers et al., 2013). Despite these insights, significant gaps persist in understanding how diatom diversity is shaped by the interplay of environmental factors, land use patterns, and geographical gradients in large, heterogeneous river systems like the Yellow River. This highlights the necessity for region-specific research to clarify the ecological roles of diatoms and their responses to multi-scale environmental variations.

While existing studies have established the significant influence of environmental variables, such as nutrient levels, dissolved oxygen, and turbidity in shaping benthic diatom communities (Tsoi et al., 2017), the interactions between these variables and spatial factors, such as geography and land use, remain poorly understood in large river systems. Rivers like the Yellow River exhibit strong environmental gradients due to their vast geographical extent and diverse land use patterns. For example, the upstream region, originating from the Qinghai-Tibet Plateau, features relatively pristine conditions, while the middle reaches region is dominated by agricultural activities and sediment inputs from the Loess Plateau. Lower reaches areas, heavily urbanized, face compounded pressures from nutrient loading, industrial discharges, and hydrological alterations such as dam construction (Zhang et al., 2018). These gradients create highly heterogeneous conditions that influence diatom diversity in complex ways. Previous research has often focused on localized or small-scale factors, neglecting the broader spatial and land use patterns that modulate community assembly processes. For instance, studies on the Yangtze River have emphasized the impacts of urbanization on diatom diversity but have not accounted for the interactive effects of geographical and environmental gradients (Liu et al., 2016). Similarly, research on other river systems has demonstrated the influence of land use on nutrient dynamics but has rarely linked these changes to diatom community responses. Recent studies on the Yellow River have highlighted the sensitivity of diatom communities to nutrient inputs and land use changes (Ren and Liu, 2024), underscoring the need for integrated analyses of multi-scale drivers. This study advances beyond previous research on large river systems by integrating environmental, geographical, and anthropogenic drivers within a single analytical framework to elucidate their combined effects on benthic diatom diversity. Unlike prior studies that often focused on isolated factors, our work employs structural equation modeling (SEM) to disentangle direct and indirect effects across multiple scales in the Yellow River, a highly sediment-laden and heterogeneous system. Furthermore, it examines region-specific dynamics in the middle and lower reaches, offering insights for tailored management strategies addressing the Yellow River basin's unique ecological challenges.

This study focuses on the middle reaches and lower reaches sections of the Yellow River, where the impacts of anthropogenic activities and environmental gradients are most pronounced. The primary objectives are to: (1) assess the influence of key environmental factors on diatom diversity, (2) evaluate the roles of land use and geography in shaping diatom community structure, and (3) explore the synergistic effects of these factors to identify mechanisms driving biodiversity patterns. The findings of this study will contribute to a deeper understanding of how environmental and anthropogenic factors jointly shape biodiversity patterns in large river systems. These insights are critical for informing evidence-based conservation strategies and sustainable river basin management, particularly in regions facing increasing human pressures such as the Yellow River.

## 2 Materials and methods

### 2.1 Study area

The Yellow River, the second longest river in China, stretches approximately 5,464 kilometers and flows through nine provinces, encompassing a drainage basin of 795,000 square kilometers. Known as the “Mother River of China”, it plays a critical role in supporting agricultural production, providing water resources, and sustaining economic development for over 100 million people (Saito et al., 2001). The river exhibits distinct geographical and environmental gradients along its course, which can be broadly divided into the upper, middle, and lower reaches. The upper reach, originating from the Qinghai-Tibet Plateau, features pristine conditions and low human activity. The middle reach, flowing through the Loess Plateau, experiences significant sediment input and moderate land use changes, including agriculture and grazing. The lower reach, heavily urbanized, is characterized by intensive agricultural runoff, industrial discharges, and hydrological modifications, such as dams and reservoirs (Wang et al., 2007). These geographical variations lead to distinct environmental conditions, such as differences in nutrient levels, turbidity, and hydrological connectivity, which influence the aquatic biodiversity along the river. The lower reaches of the Yellow River are characterized by a unique “suspended river” feature, where the riverbed is elevated above the surrounding floodplain due to heavy sediment deposition, influencing nutrient and pollutant transport dynamics (Parr et al., 2004).

**Figure 1:**
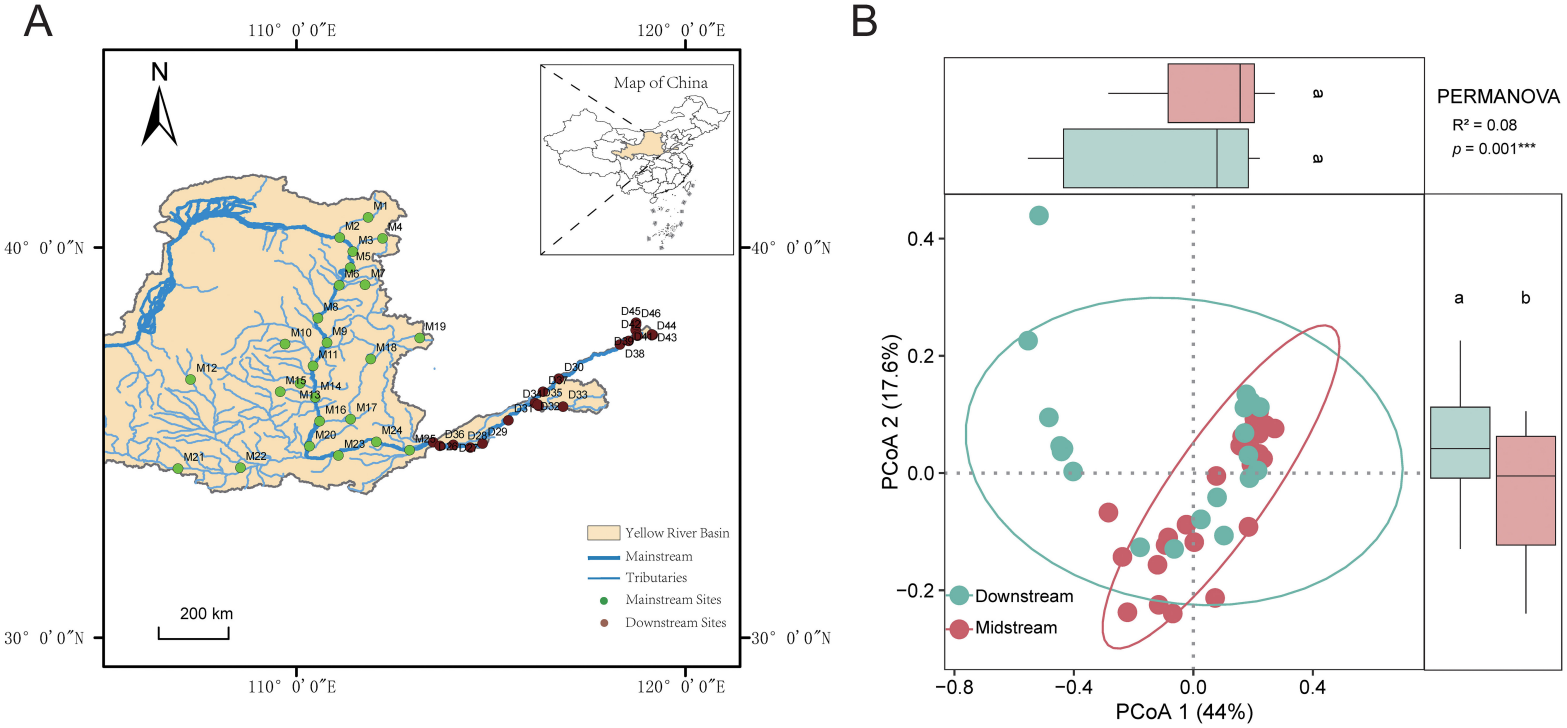
**(A)** The location of Yellow River Basin and distribution of sampling sites. **(B)** The results of PCoA, ANOVA of PCoA1 and PCoA2, and PERMANOVA significance based on the Euclidean distance of factors. Different letters indicate significant differences. ***p = 0.001.

This study focuses on the middle reaches and lower reaches sections of the Yellow River, where the impacts of human activities and environmental gradients are most significant. A total of 46 sampling sites were established along the main channel and tributaries of the river, with 25 sites in the middle reaches region and 21 in the lower reaches region (Figure 1A, Supplementary Table S1). Benthic diatoms and water samples were collected in June 2023. This study focuses on the middle and lower reaches of the Yellow River, where anthropogenic activities and environmental gradients are most pronounced, exerting significant pressures on aquatic biodiversity. The upper reach, characterized by relatively pristine conditions and lower human impact, was excluded due to logistical constraints and its limited relevance to the study's focus on human-induced ecological changes.

### 2.2 Field sampling and processing

Benthic diatoms were collected from natural substrata, including stones. The samples were brushed from the surface of substrates under 10–30 cm of water using toothbrushes. A composite sample was collected by mixing samples from three locations at each site, and rinsed several times with distilled water before being placed into 100 mL plastic bottle, and preserved with 5% formalin. The organic material of the diatom samples was removed using concentrated nitric acid and a microwave-accelerated reaction system (Parr et al., 2004). The rest diatom valve samples were divided into two subsamples, one for measuring the abundance using a light microscope (Nikon Ci-L, Tokyo, Japan), and the other for identifying valves striae using a scanning electron microscope (JEOL JSM-7800F, Tokyo, Japan). Permanent slides were prepared using Naphrax (Brunel Microscopes, Chippenham, UK) and were observed under the light microscope at 1,000 × magnification. At least 500 diatom valves were counted for each sample. Samples for scanning electron microscope were coated with gold in a sputter-coater (Kling and Håkansson, 1988). Photographs from both the light microscope and scanning electron microscope were combined for identification, and all specimens were identified at the species level. The diatom taxa were identified according to Krammer and Lange-Bertalot (Round, 1990), and Diatoms of North America (http://diatoms.org/).

### 2.3 Environmental variables

A multiparameter water quality analyzer was used on-site to measure parameters such as water temperature, electrical conductivity, dissolved oxygen, and pH. Additional physicochemical factors, including chemical oxygen demand (COD_Mn_), total nitrogen (TN), total phosphorus (TP), ammonia nitrogen (NH3–N), and turbidity, were analyzed in accordance with the Chinese National Water Quality Standards and guidelines from the United States Environmental Protection Agency (U.S. Environmental Protection Agency, 2012). To investigate the impact of human disturbances within the watershed, land use data for the upstream areas of each sampling site were obtained using Landsat imagery processed through ArcGIS v10.3 (ESRI Inc., Redlands, CA, USA). Land use data were derived from Landsat 8 imagery (30 m resolution, acquired in summer 2022) processed by the authors using ArcGIS v10.3 (ESRI Inc., Redlands, CA, USA). Images with <10% cloud cover were selected from June–August to capture peak vegetation signals. Land use was classified into seven categories (Urban, Crop, Ranch, Vegetation, Water, Tree and Bare) using a supervised maximum likelihood classification, with an overall accuracy of 92% based on ground-truth validation with 200 reference points. These data, alongside water quality parameters (e.g., TN, TP, turbidity), are provided in Supplementary Data Sheet 1.

### 2.4 Data analysis

Environmental variables of different habitats were analyzed by principal coordinate analysis (PCoA) based on Euclidean distance matrix. Prior to PCoA, environmental variables were standardized to ensure comparability. To examine the determinants of benthic diatom diversity, multiple linear regression models were developed for alpha diversity indices. Predictor variables included water quality parameters, geographical variables (longitude and latitude), and land use proportions (Urban, Crop, Ranch, Vegetation, Water, Tree, Bare) (Gelis et al., 2024). Variance inflation factors (VIFs) were calculated to assess multicollinearity, with a threshold of VIF <5 used to retain predictors, ensuring independence among variables. A backward stepwise selection procedure was applied to exclude non-significant predictors (*p* > 0.05), optimizing model fit. Model assumptions, including normality (Shapiro-Wilk test) and homoscedasticity (Breusch-Pagan test), were verified to ensure statistical robustness (Niu et al., 2024).

Beta diversity was partitioned into its turnover and nestedness components using the Bray-Curtis dissimilarity index to quantify spatial variation in community composition. Variance partitioning analysis (VPA) was performed to evaluate the relative contributions of environmental, geographical, and land use variables to beta diversity. Taxonomic alpha diversity (AlphaTD) was quantified using Shannon diversity, species richness, and evenness indices, while taxonomic beta diversity (BetaTD) was assessed using the Bray-Curtis dissimilarity index, partitioned into turnover and nestedness components (Villéger et al., 2013). Structural Equation Modeling (SEM) was employed to disentangle the direct and indirect effects of these variables on diatom community composition and diversity. The SEM approach allowed for the integration of causal relationships among environmental drivers and land use, providing a comprehensive understanding of their interactions. The SEM framework was constructed based on ecological theory, hypothesizing that geographical factors influence land use patterns, which in turn affect water quality parameters, ultimately shaping diatom diversity (Silva et al., 2019; Desrosiers et al., 2013). This causal pathway was informed by prior studies demonstrating the hierarchical influence of spatial and anthropogenic factors on aquatic communities.

All statistical analyses were performed using R software (v4.2.0), with packages such as “vegan” for diversity analysis (Oksanen et al., 2022), “sem” for structural equation modeling, and “FactoMineR” for PCoA. Maps of land use and sampling sites were generated in ArcGIS v10.3.

## 3 Results

### 3.1 Environmental variability

The results of PCoA analysis indicated significant differences in environmental factors between the middle and lower reaches of the Yellow River (Figure 1B, Supplementary Figure S1). Specifically, chemical oxygen demand (COD) and ammonia nitrogen (NH3–N) concentrations were significantly higher in the lower reaches compared to the middle reaches, as were total phosphorus (TP) and total nitrogen (TN) levels. Water temperature (T) was higher in the lower reaches, while dissolved oxygen (DO) was more abundant in the middle reaches. Conductivity (Cond) was also higher in the lower reaches. Regarding land use, the middle reaches were primarily dominated by vegetation cover, while the lower reaches featured extensive urban and agricultural landscapes. No significant differences were observed between tributaries and the main channel (Supplementary Figure S3A).

### 3.2 Diatom community composition

A total of 130 species of benthic diatoms were identified in the middle and lower reaches of the Yellow River, belonging to 2 classes, 6 orders, 10 families, and 40 genera. At the family level, benthic diatoms were primarily composed of Cymbellaceae, Gomphonemaceae, Naviculaceae, and Nitzschiaceae. Naviculaceae was the most dominant family, accounting for 33.08%, followed by Nitzschiaceae, which accounted for 17.70%.

**Figure 2:**
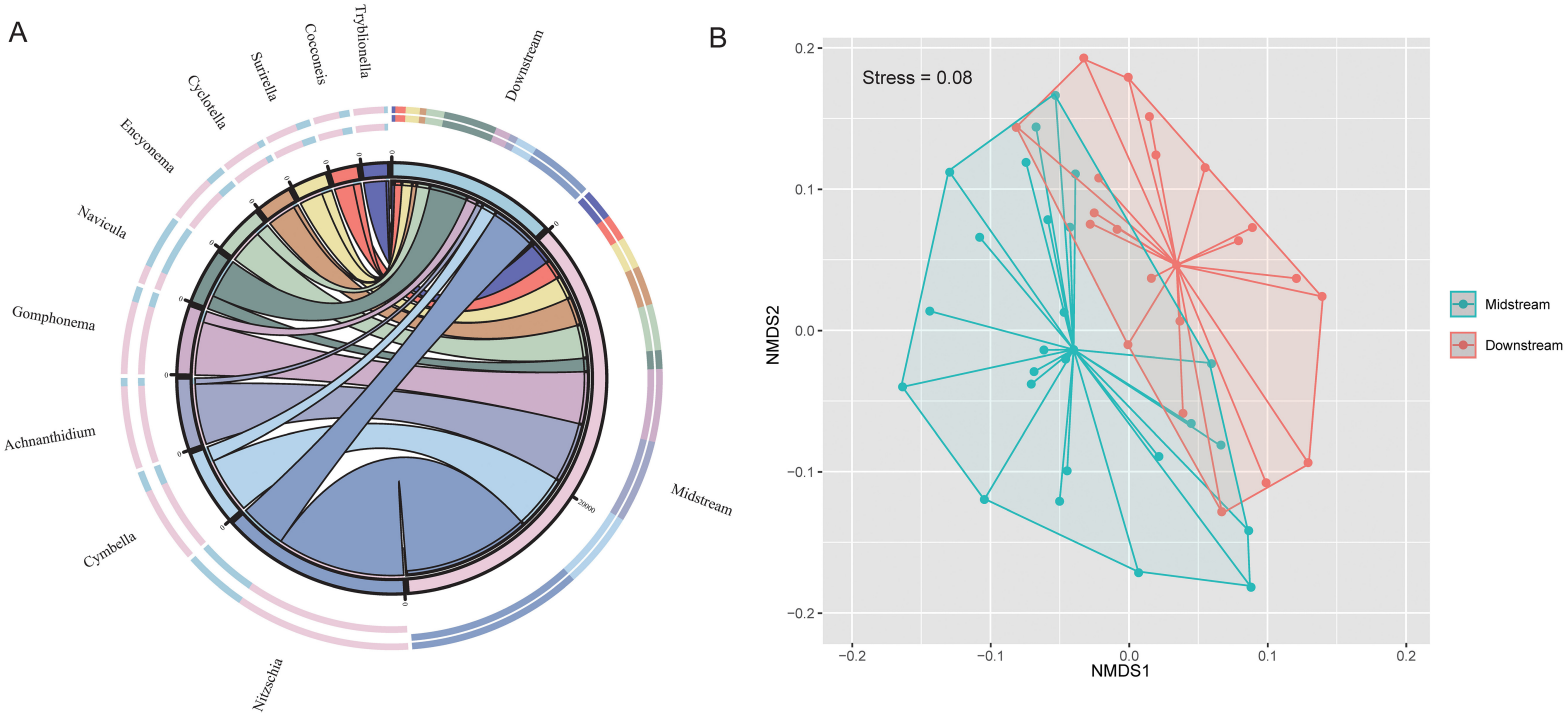
**(A)** Chord diagram showing the distribution of dominant benthic diatom genera between the middle reaches and lower reaches Yellow River. **(B)** NMDS ordination plot illustrating distinct diatom community structures between the two regions (stress = 0.08).

The diatom species composition showed distinct variations between the middle and lower reaches of the Yellow River (Supplementary Figure S2). The NMDS results indicate significant differences in the benthic diatom community structure between the middle and lower reaches of the Yellow River (Figure 2B). The genera *Nitzschia, Navicula*, and *Cyclotella* dominated in the lower reaches, with significantly higher abundances compared to the middle reaches. In contrast, genera such as *Cymbella, Achnanthidium*, and *Gomphonema* were more abundant in the middle reaches. Other genera, including *Encyonema, Surirella, Cocconeis*, and *Tryblionella*, exhibited lower overall abundances but varied in distribution patterns across the two regions (Figure 2A). These differences reflect the spatial variability in environmental conditions along the river. Notably, diatom community composition showed no significant differences between main channel and tributary sites (*p* > 0.05; Supplementary Figures S3B, S4).

### 3.3 Alpha and beta diversity

**Figure 3:**
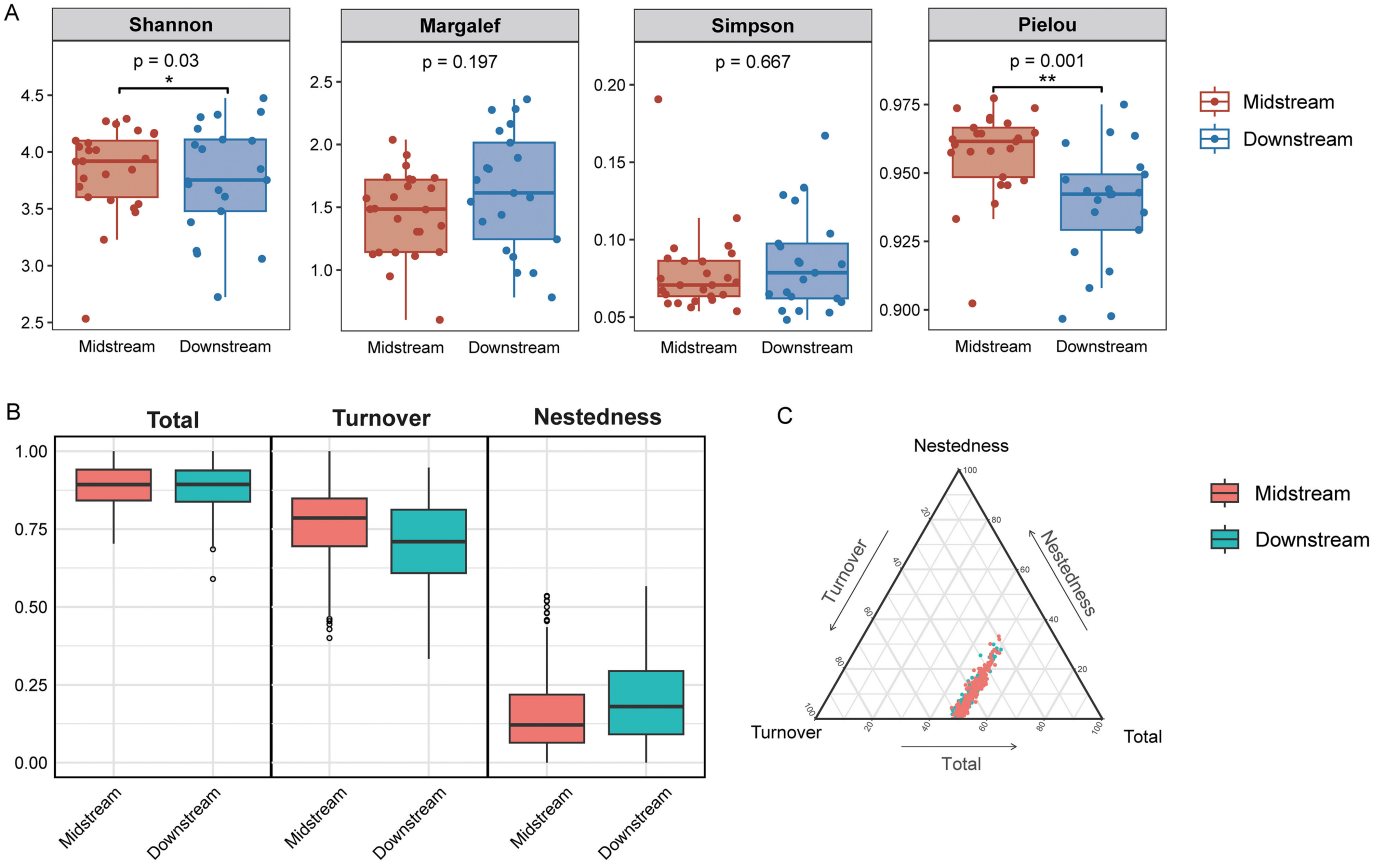
Diversity analysis of benthic diatom communities in the middle and lower reaches of the Yellow River. **(A)** Boxplots of α diversity indices for middle reaches and lower reaches regions. **(B)** Boxplots of β diversity components for both regions. **(C)** Ternary plot visualizing the relative contributions of turnover (species replacement between sites), nestedness (species loss or subset patterns), and total beta diversity across all samples in the middle and lower reaches of the Yellow River. *p < 0.05, **p < 0.01.

The alpha diversity of benthic diatom communities in the middle reaches and lower reaches sections of the Yellow River revealed significant regional differences (Figure 3A). Shannon diversity index was significantly higher in the middle reaches compared to the lower reaches (*p* < 0.05), indicating greater species diversity in the middle reaches region. Similarly, Pielou's evenness index also showed significantly higher values in the middle reaches (*p* < 0.05), reflecting more uniform species distributions. However, no significant differences were observed for the Margalef richness index (*p* > 0.05) or Simpson diversity index (*p* > 0.05) between the two regions, suggesting that species richness and dominance patterns were relatively consistent.

Beta diversity was analyzed to explore spatial differences in community composition of benthic diatoms between the middle reaches and lower reaches regions of the Yellow River (Figures 3B, C). The results revealed that total beta diversity was consistently high in both regions, indicating notable dissimilarities in community composition across sampling sites. The partitioning of beta diversity into turnover (β Turnover) and nestedness (β Nestedness) components showed that turnover was the primary driver of beta diversity, while the contribution of nestedness was relatively minor. In the middle reaches region, the turnover component (β Turnover) dominated beta diversity, highlighting significant species replacement between sampling sites. This suggests that the spatial heterogeneity of diatom communities in the middle reaches is mainly attributed to the replacement of species along environmental gradients. In contrast, while turnover was also the major contributor to beta diversity in the lower reaches region, the nestedness component (β Nestedness) contributed slightly more than in the middle reaches. This indicates that species loss or sharing played a relatively larger role in shaping community composition in the lower reaches area. The ternary plot illustrated the proportional contributions of turnover, nestedness, and total beta diversity across all samples. Clear clustering patterns were observed, with samples from the middle reaches and lower reaches regions forming distinct groups. Middle reaches samples were more tightly clustered, suggesting more consistent spatial patterns in species composition, whereas lower reaches samples displayed greater variation, reflecting increased community heterogeneity.

### 3.4 Multiple factors affecting biodiversity

**Figure 4:**
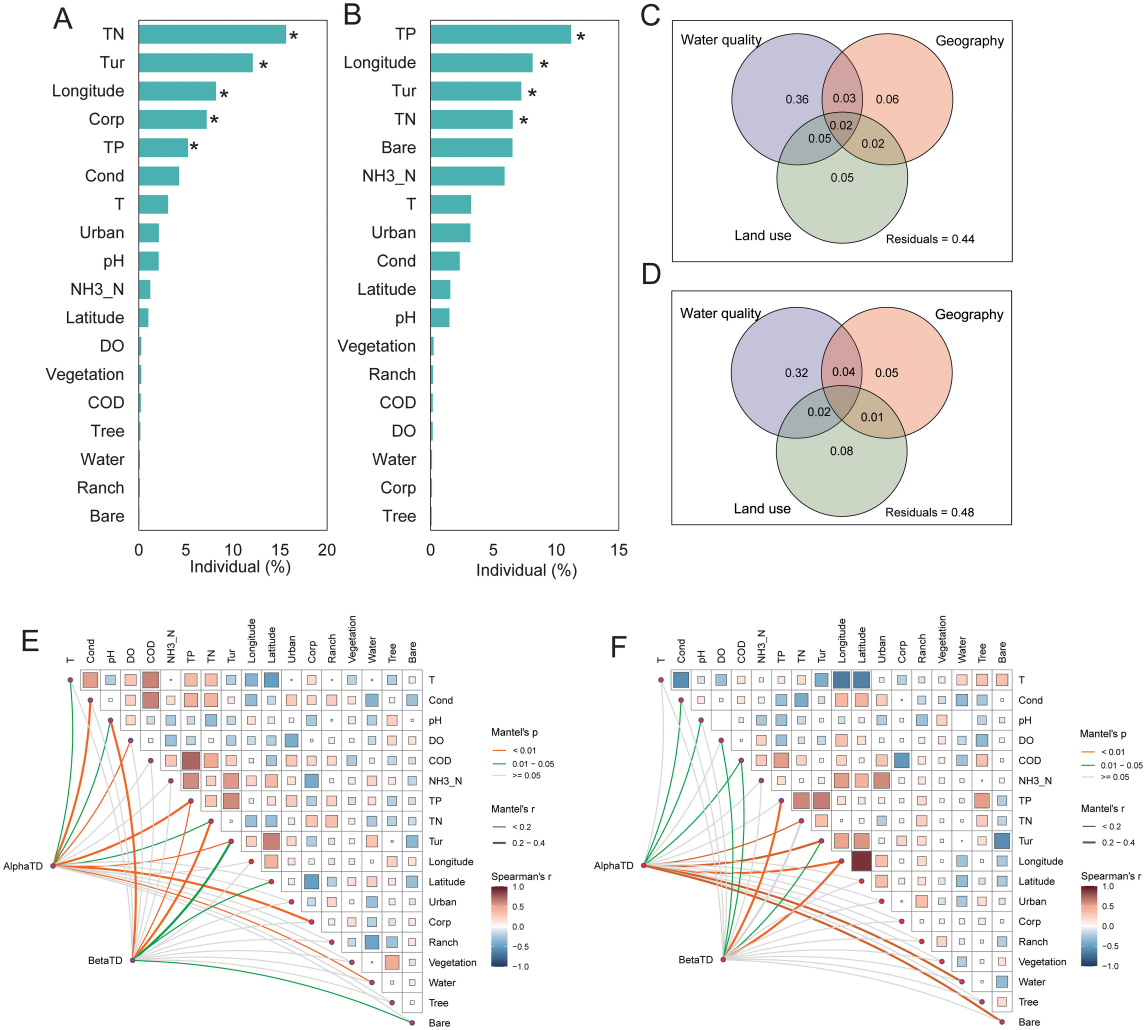
Key environmental and spatial drivers of benthic diatom diversity in the middle **(A)** and lower **(B)** reaches of the Yellow River. Contributions of environmental, geographical, and land use variables to alpha and beta diversity, where turnover refers to species replacement and nestedness indicates species loss or subset patterns. Variance partitioning for middle **(C)** and lower **(D)** reaches illustrates their relative explanatory power. Correlation and Mantel test results **(E, F)** show relationships between diversity and variables. *p < 0.05.

The contributions of environmental, geographical, and land use factors to benthic diatoms in the middle and lower reaches of the Yellow River were evaluated using barplots and variance partitioning (Figures 4A, B). In the middle reaches, total nitrogen (TN), turbidity (Tur), longitude, and total phosphorus (TP) were identified as the most significant predictors. Among these, TN and Turbidity had the highest explanatory power, indicating that nutrient enrichment and water clarity strongly influence species richness and evenness. Similarly, for lower reaches, TP emerged as the strongest predictor, followed by longitude, TN, and Turbidity (*p* < 0.05).

The variance partitioning analysis further illustrates these regional differences in explanatory factors (Figures 4C, D). In the middle reaches, water quality accounted for 36% of the explained variation in diatom diversity, followed by geography (6%) and land use (5%), with residuals contributing 44%, indicating additional unmeasured factors influencing diversity in this region. In the lower reaches, the contribution of water quality decreased to 32%, while the influence of land use increased to 8%, and geography remained relatively stable at 5%. Residuals accounted for 48% of the unexplained variation, suggesting increased environmental complexity and a greater role of unmeasured factors in the lower reaches compared to the middle reaches.

The Mantel test and correlation analysis identified significant associations between benthic diatom diversity and environmental variables in both the middle reaches and downstream sections of the Yellow River (Figure 4). In the middle reaches, alpha diversity was strongly correlated with total nitrogen (TN), turbidity (Tur), and total phosphorus (TP), with Mantel's *p* < 0.01 indicating significant relationships. Beta diversity was similarly associated with TN, Tur, and TP. Geographical factors, such as longitude, also showed moderate correlations with diversity metrics. In the lower reaches, alpha diversity was significantly related to total phosphorus (TP), turbidity (Tur), and total nitrogen (TN), with Mantel's *p* < 0.01 for these variables. Beta diversity exhibited similar associations with TP and Tur, while geographical factors, such as longitude and latitude, showed weaker but notable correlations. Land use variables, including urban and agricultural coverage, displayed increased correlations with both alpha diversity and beta diversity compared to the middle reaches. Nutrient-related variables (TP, TN) and turbidity were consistently significant across both regions, while the relative importance of these factors varied between the middle reaches and lower reaches sections. Geographical and land use factors exhibited region-specific patterns of association with diversity metrics.

**Figure 5:**
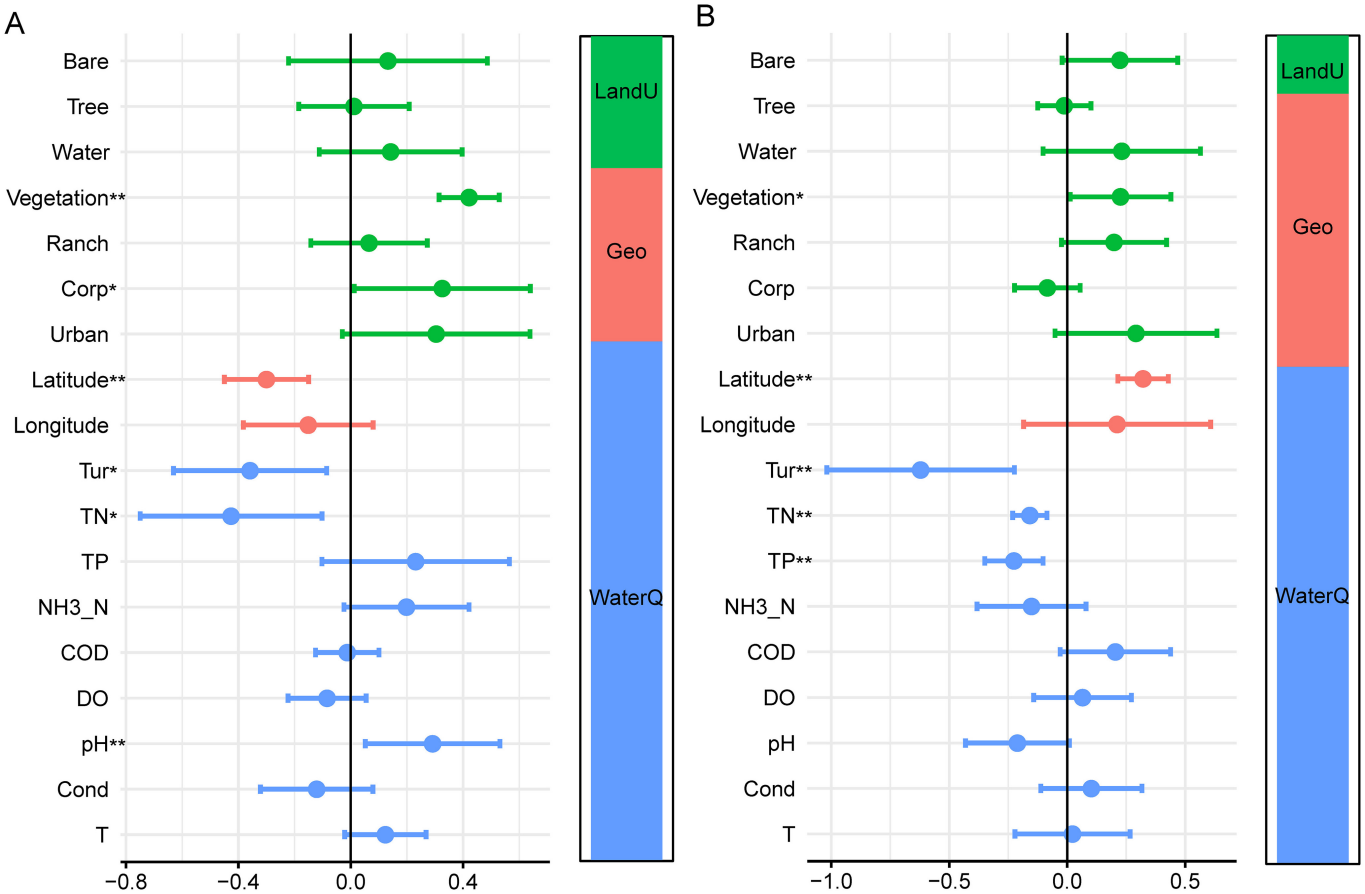
The relative influence of multiple factors on diatom communities in the middle **(A)** and lower **(B)** reaches of the Yellow River, *p < 0.05, **p < 0.01.

The regression analysis and variance partitioning further clarified the contributions of individual predictors and variable categories (water quality, geography, and land use) to diatom diversity (Figure 5). The results of the analysis demonstrate differences in the key drivers of benthic diatom communities between middle reaches (Figure 5A) and lower reaches (Figure 5B) regions. In the middle reaches region, water quality factors such as turbidity (Tur), total nitrogen (TN), and total phosphorus (TP) were the primary contributors, with significant positive and negative associations (*p* < 0.05), indicating their strong influence on diatom community composition. Geographical factors, such as latitude, also had a significant impact, though to a lesser extent, while land-use factors, including vegetation and corporate land, were moderately influential. The variance partitioning analysis revealed that water quality accounted for the largest proportion of explained variation, followed by land use and geography. In the lower reaches region, the influence of water quality factors remained dominant, with TP, TN, and Turbidity showing highly significant effects (*p* < 0.01). However, the contribution of land-use factors, including vegetation, became more prominent, reflecting the increasing role of anthropogenic influences in this region. Geographical factors, particularly latitude, maintained a significant, albeit smaller, effect compared to middle reaches. The variance partitioning analysis showed a consistent dominance of water quality in explaining diatom community variation, though the role of land use increased lower reaches, highlighting spatial and environmental variability between the two regions.

### 3.5 Pathway analyze for impact of multiple factors on biodiversity

**Figure 6:**
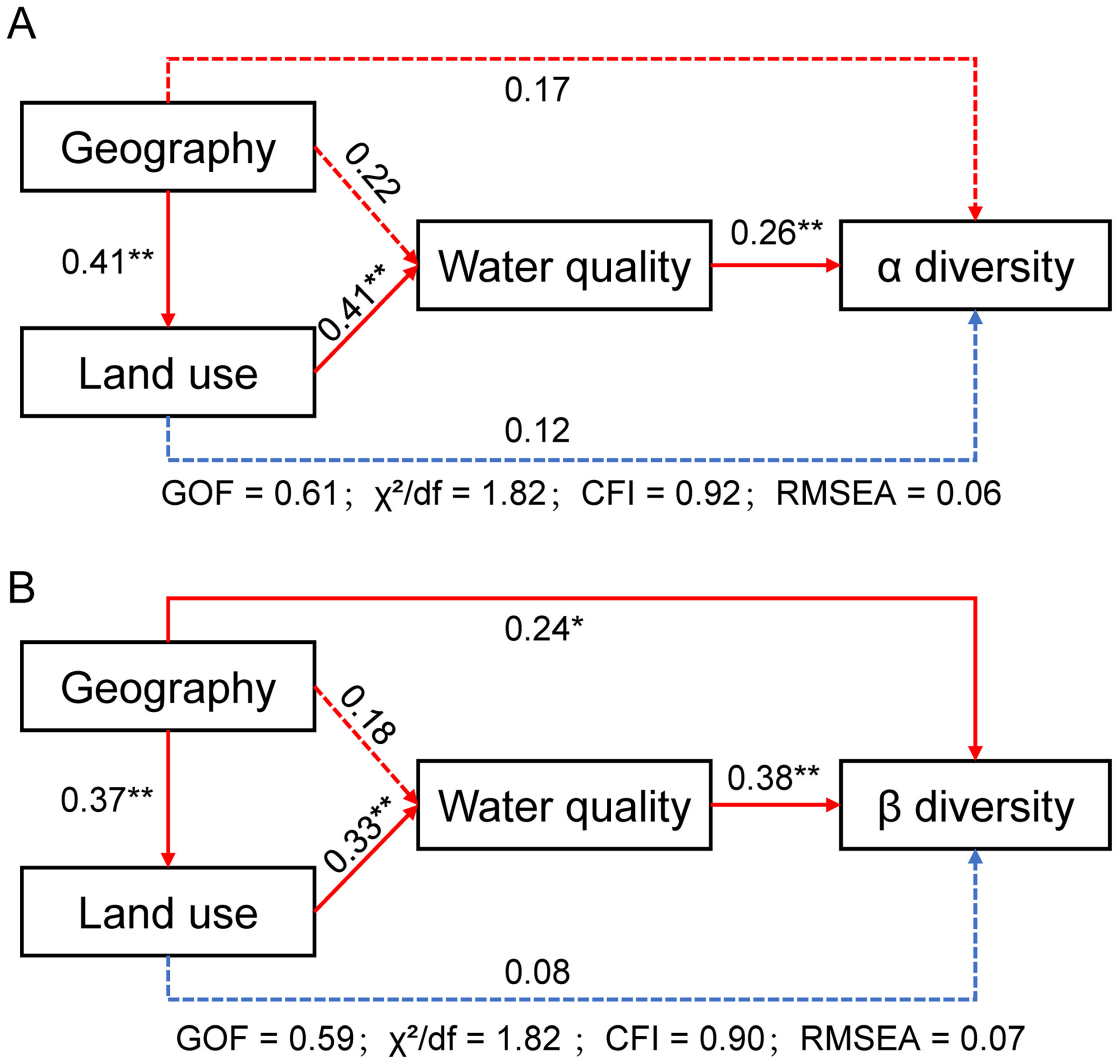
Structural equation models (SEMs) for Yellow River basin based on the relationship between geography, land use and water quality and diversity. Solid lines indicate significant paths (*p < 0.05, **p < 0.01, ***p < 0.001); dotted lines mean non-significant paths. Orange lines show positive path strengths and blue lines represent negative path strengths. **(A)** Alpha diversity; **(B)** Beta diversity.

The structural equation modeling (SEM) analysis revealed the direct and indirect effects of geography, land use, and water quality on alpha and beta diversity of benthic diatoms (Figure 6). For alpha diversity (Figure 6A), water quality exhibited a significant direct positive effect (path coefficient = 0.26, *p* < 0.01), while geography and land use had indirect influences mediated through water quality. Geography significantly affected land use (path coefficient = 0.41, *p* < 0.01) and water quality (path coefficient = 0.22, *p* < 0.05), indicating that spatial factors contribute to changes in environmental and land use characteristics. Land use also had a strong direct effect on water quality (path coefficient = 0.41, *p* < 0.01). The model fit was assessed using multiple indices: goodness-of-fit (GOF = 0.61), χ^2^/d*f* = 1.82, Comparative Fit Index (CFI = 0.92), and Root Mean Square Error of Approximation (RMSEA = 0.06). For beta diversity (Figure 6B), water quality showed a significant direct positive effect (path coefficient = 0.38, *p* < 0.01), and geography had a direct effect (path coefficient = 0.24, *p* < 0.05). Geography also influenced beta diversity indirectly through land use (path coefficient = 0.37, *p* < 0.01) and water quality (path coefficient = 0.33, *p* < 0.01). The beta diversity model fit was satisfactory (GOF = 0.59, χ^2^/d*f* = 1.95, CFI = 0.90, RMSEA = 0.07). Path selection was based on ecological theory, prioritizing variables with significant correlations in preliminary analyses. Alternative models, including direct paths from geography to diversity, were tested but yielded poorer fit, confirming the robustness of the reported models.

## 4 Discussion

This study provided a comprehensive analysis of the factors influencing benthic diatom diversity in the middle and lower reaches of the Yellow River, integrating environmental, geographical, and land-use variables to uncover their direct and indirect effects. By combining structural equation modeling with alpha and beta diversity metrics, the research offered new insights into the interplay between natural and anthropogenic drivers of biodiversity in a highly dynamic and sediment-rich river system. Unlike previous studies that often focused on isolated drivers or specific river segments, our study adopts a holistic, multi-scale approach tailored to the Yellow River's unique environmental gradients and human-induced pressures. This framework not only elucidates region-specific patterns, such as the dominance of eutrophic taxa in the lower reaches, but also provides insights for understanding biodiversity dynamics in other large, human-impacted river systems worldwide.

### 4.1 Spatial patterns of diatom communities in the middle and lower Yellow River

The spatial distribution of diatom communities in the Yellow River reveals significant differences between the middle and lower reaches, driven by both natural gradients and anthropogenic pressures. In the middle reaches, higher alpha diversity, as indicated by metrics such as the Shannon diversity index and evenness, is associated with the predominance of agricultural and natural vegetation landscapes (Figure 4). Genera such as *Cymbella, Gomphonema*, and *Achnanthidium* dominate in this region, indicative of conditions with moderate nutrient levels and relatively stable hydrology (Silva et al., 2019; Wang et al., 2021). These taxa are often linked to mesotrophic or oligotrophic waters, conditions typically found in environments with limited human impact (B-Béres et al., 2016). Their presence underscores the importance of maintaining stable and moderate environmental conditions to sustain biodiversity. Conversely, the lower reaches of the Yellow River, heavily impacted by urbanization and industrial activity, showed distinct diatom community compositions. Genera such as *Nitzschia, Navicula*, and *Cyclotella* dominate in this region, reflecting eutrophic conditions characterized by high nutrient loads and turbidity (Lange et al., 2011). This shift in community structure is consistent with previous studies in other large river systems, such as the Yangtze River (Chen et al., 2022; Atti et al., 2023), where similar taxa dominated under high anthropogenic pressure (Chrea et al., 2024). The spatial variability in beta diversity, particularly the dominance of species turnover over nestedness, further reflects the ecological heterogeneity caused by environmental gradients and human influences (Figure 3). While species turnover remains the primary component of beta diversity in both reaches, the slightly higher contribution of nestedness in the lower reaches highlights species loss and community homogenization due to severe habitat degradation. This increase in nestedness indicates systematic species loss, where less tolerant taxa are replaced by eutrophic specialists under high nutrient loads and turbidity, signaling ecological degradation (Wang et al., 2021). This observed spatial differentiation aligns with findings from other studies on large river systems. For example, Lacroix et al. (2007) reported similar shifts in diatom communities along environmental gradients in the Rhine River. The clustering of diatom communities in the middle reaches, as seen in NMDS and PCoA analyses (Figure 1; Figure 2B), indicates greater consistency in environmental conditions compared to the lower reaches. This highlights the importance of preserving these relatively stable sections to maintain ecological integrity across the river system.

### 4.2 Environmental factors influencing diatom communities

Environmental factors, including nutrient levels, turbidity, and dissolved oxygen, drove benthic diatom diversity in the Yellow River, with distinct regional patterns. In the middle reaches, moderate TN and turbidity supported higher alpha diversity, fostering stable communities of taxa like Navicula and Gomphonema under consistent hydrological conditions (Figure 4), consistent with findings on nutrient-driven diatom stability in northeast China's rivers (Smeti et al., 2023). Conversely, the lower reaches exhibited elevated TP and turbidity, intensified by urbanization and agricultural runoff, promoting eutrophic taxa like Nitzschia and community homogenization (Figure 5). Recent studies in the Yellow River Delta confirm that seasonal nutrient enrichment, particularly TP, shifts diatom composition toward eutrophic dominance (Zhao et al., 2025). These patterns align with environmental filtering mechanisms in nutrient-enriched systems (Aarnio and Soininen, 2024). The Yellow River's water-sediment dynamics, amplified by anthropogenic inputs like industrial discharges, exacerbated nutrient loading and habitat degradation (Zhang et al., 2024a; Zhang and Pan, 2024). Land-use changes, notably urbanization in the lower reaches, further reduced biodiversity by increasing salinity and nutrient inputs, as seen in coastal wetland restoration impacts on diatom assemblages (Martin-Devasa et al., 2024). These contrasts highlight the distinct ecological dynamics within the Yellow River basin, where the middle reaches benefit from relatively balanced nutrient inputs and less severe human interference, supporting more stable diatom communities, while the lower reaches face greater variability and degradation due to intensified anthropogenic activities. In the lower reaches, the Yellow River's “suspended river” morphology, where the riverbed is elevated above the floodplain (Parr et al., 2004), complicates nutrient and pollutant transport from adjacent land use to the main channel. Runoff from urban and agricultural areas primarily enters the river through drainage channels, irrigation returns, and occasional flood events that breach levees, delivering nutrients and sediments that increase turbidity.

Variance partitioning showed environmental factors explaining only 32–36% of diatom variation, with 44–48% residuals (Figures 4C, D), indicating stochastic processes in assembly. In the Yellow River, hydrological disturbances like flood discharges and water-sediment regulation promote dispersal limitation and ecological drift, disrupting connectivity and causing random species loss, especially in the lower reaches. Recent studies confirm this: stochastic dominance in fragmented stream diatom metacommunities (Martin-Devasa et al., 2024) and seasonal drift in phytoplankton under flow fluctuations (Hu et al., 2022). These mechanisms complement deterministic drivers, advancing neutral theory applications in sediment-laden rivers.

### 4.3 The combined impact of multiple environmental factors and ecological significance

The combined effects of environmental, geographical, and land use factors on diatom communities reveal complex interactions that shape biodiversity patterns in the Yellow River. Structural equation modeling (SEM) highlighted the direct impact of water quality on alpha and beta diversity, with geography and land use exerting indirect effects through changes in environmental conditions (Figure 6). This underscores the critical role of water quality as a mediator of biodiversity patterns in river systems (Lange et al., 2011). The Yellow River is renowned for its high sediment load and complex hydrological conditions, with the middle reaches region's elevated turbidity and moderate nutrient inputs further amplifying the role of water quality in shaping diatom diversity. In the middle reaches, water quality variables such as TN and turbidity were the most significant predictors of diatom diversity, reflecting the direct impact of nutrient enrichment and sediment transport (Chrea et al., 2024). The observed relationships between alpha diversity and nutrient levels indicate that moderate nutrient enrichment supports higher diversity, while excessive inputs may lead to ecological imbalances. In the lower reaches, the increasing importance of land use factors, such as urban and agricultural coverage, highlights the compounded effects of human activity on diatom communities. These changes reflect the broader ecological impacts of urbanization and industrialization, including altered hydrological regimes, increased nutrient loading, and habitat fragmentation. Similar patterns have been reported in other heavily modified river systems, where anthropogenic pressures resulted in community homogenization and biodiversity loss (Vanhoutte et al., 2006; Sun et al., 2018).

The ecological significance of diatom communities extends beyond their role as bioindicators (Minaoui et al., 2021). As primary producers, they play critical roles in nutrient cycling (Chai et al., 2021), food web dynamics, and sediment stabilization, contributing to the overall functioning of aquatic ecosystems. The observed shifts in community composition and diversity in the Yellow River have implications for ecosystem services, including water purification and biodiversity support. Understanding the combined effects of environmental, geographical, and land use factors is essential for developing integrated conservation and management strategies that address the multi-scale challenges facing large river systems. In conclusion, this study highlights the complex interactions between environmental, geographical, and anthropogenic factors that shape diatom diversity in the Yellow River. The findings underscore the importance of preserving ecological integrity through targeted interventions that address the unique challenges of the middle and lower reaches. Future research should explore the long-term impacts of climate change, hydrological alterations, and land use transitions on diatom communities to inform sustainable river basin management.

## 5 Conclusion

This study elucidated the factors influencing benthic diatom diversity in the Yellow River, addressing three key scientific questions. First, water quality factors (total nitrogen, total phosphorus, turbidity) were critical drivers, with middle reaches supporting higher diversity due to moderate nutrient levels, while lower reaches showed eutrophic taxa dominance due to nutrient enrichment. Second, land use and geography shaped community structure, with agricultural and vegetative landscapes supporting diverse assemblages in the middle reaches, and urban/industrial impacts causing biodiversity loss in the lower reaches. Third, water quality mediated the interplay of geography and land use, driving biodiversity patterns. These findings underscore the need for region-specific management strategies. In the middle reaches, maintaining hydrological stability through controlled water releases and moderate nutrient inputs via precision agriculture can sustain diatom diversity. In the lower reaches, reducing urban and industrial discharges through stricter wastewater treatment regulations and mitigating agricultural runoff with buffer zones are critical to prevent community homogenization and biodiversity loss. These targeted interventions, integrated with land use planning, are essential for sustainable management of the Yellow River and similar large river systems.

## Data Availability

The raw data supporting the conclusions of this article will be made available by the authors, without undue reservation.
