# Correction: Spatial pattern and environmental determinants of benthic diatom diversity in the middle and lower reaches of the Yellow River

**DOI:** 10.3389/fmicb.2025.1744178

**Published:** 2025-12-04

**Authors:** Qingyang Guo, Qin Zhu, Pengfei Peng, Qiuling Zhou, Yong Ding, Zhenchang Zhu

**Affiliations:** 1 Guangdong Basic Research Center of Excellence for Ecological Security and Green Development, Guangdong Provincial Key Laboratory of Water Quality Improvement and Ecological Restoration for Watersheds, Guangdong University of Technology, Guangzhou, China; 2 Southern Marine Science and Engineering Guangdong Laboratory (Guangzhou), Guangzhou, China; 3 Guangdong Provincial Observation and Research Station for Social-Natural Complex Ecosystems in Haizhu Wetlands, Guangzhou, China; 4 South China Sea Marine Survey Center, Ministry of Natural Resources, Guangzhou, China; 5 Central Cycleecological Technology Co., Ltd., Guangzhou, China

**Keywords:** Yellow River, benthic diatoms, biodiversity, water quality, land use

In the published article, there was an error in the **Funding** statement. The grant number for the Guangdong Introducing Innovative and Entrepreneurial Teams R&D program was given as “2019ZT08L213”. The correct Funding statement appears below.

“The author(s) declare that financial support was received for the research and/or publication of this article. The R&D program of Guangdong Provincial Department of Science and Technology (2024B1212040004).”

In the published article, there was an error in the Correspondence. The email address for the corresponding author was given as “zhenchangzhu@163.com”. The correct email address is “zhenchang.zhu@gdut.edu.cn”.

The original version of the article has been updated.

