# Supplementary material for: Spatial pattern and environmental determinants of benthic diatom diversity in the middle and lower reaches of the Yellow River
Source: Front Microbiol. 2025 Oct 16;16:1677888. doi: 10.3389/fmicb.2025.1677888 (PMC12571835; doi:10.3389/fmicb.2025.1677888)
Supplement: Supplementary file 1 [file Supplementary_file_1.docx]

# Spatial pattern and environmental determinants of benthic diatom diversity in the middle and lower reaches of the Yellow River

Qingyang Guo^a,b.c^, Qin Zhu^b^, Pengfei Peng^d^, Qiuling Zhou^e^, Yong Ding^a^, Zhenchang Zhu^a,b,c*^

^a^*Guangdong Basic Research Center of Excellence for Ecological Security and Green Development, Guangdong Provincial Key Laboratory of Water Quality Improvement and Ecological Restoration for Watersheds, Guangdong University of Technology, Guangzhou, 510006, China*

^b^*Southern Marine Science and Engineering Guangdong Laboratory (Guangzhou), Guangzhou, 511458, China*

^c^*Guangdong Provincial Observation and Research Station for Social-Natural Complex Ecosystems in Haizhu Wetlands, Guangzhou, 510310, China*

^d^*South China Sea Marine Survey Center, Ministry of Natural Resources, Guangzhou 510300, China*

^e^*Central Cycleecological Technology Co., Ltd. Guangzhou, 510655, China*

Tab. S1 Sampling sites distribution

| Code | Name | Reaches | River Class | Longitude | Latitude |
| --- | --- | --- | --- | --- | --- |
| 1 | M1 | Midstream | Tributary | 111.8388556N | 40.77278 |
| 2 | M2 | Midstream | Mainstream | 111.1107389 | 40.25861 |
| 3 | M3 | Midstream | Mainstream | 111.4533831 | 39.90194 |
| 4 | M4 | Midstream | Tributary | 112.2100517 | 40.23028 |
| 5 | M5 | Midstream | Mainstream | 111.3819992 | 39.48778 |
| 6 | M6 | Midstream | Mainstream | 111.0964639 | 39.03083 |
| 7 | M7 | Midstream | Tributary | 111.7674697 | 39.04528 |
| 8 | M8 | Midstream | Mainstream | 110.5539467 | 38.18861 |
| 9 | M9 | Midstream | Mainstream | 110.782375 | 37.56056 |
| 10 | M10 | Midstream | Tributary | 109.7116175 | 37.53194 |
| 11 | M11 | Midstream | Mainstream | 110.4254558 | 36.96083 |
| 12 | M12 | Midstream | Mainstream | 107.2833144 | 36.61806 |
| 13 | M13 | Midstream | Tributary | 110.0828133 | 36.51833 |
| 14 | M14 | Midstream | Mainstream | 110.4825 | 36.16139 |
| 15 | M15 | Midstream | Tributary | 109.5830556 | 36.30417 |
| 16 | M16 | Midstream | Mainstream | 110.5966667 | 35.5475 |
| 17 | M17 | Midstream | Tributary | 111.3961111 | 35.60444 |
| 18 | M18 | Midstream | Tributary | 111.91 | 37.14639 |
| 19 | M19 | Midstream | Tributary | 113.1663889 | 37.68889 |
| 20 | M20 | Midstream | Mainstream | 110.3397222 | 34.905 |
| 21 | M21 | Midstream | Tributary | 106.9561111 | 34.33389 |
| 22 | M22 | Midstream | Tributary | 108.555 | 34.3625 |
| 23 | M23 | Midstream | Mainstream | 111.0819444 | 34.67639 |
| 24 | M24 | Midstream | Mainstream | 112.0527778 | 35.01917 |
| 25 | M25 | Midstream | Mainstream | 112.9094444 | 34.805 |
| 26 | D26 | Downstream | Mainstream | 113.5158333 | 35.00806 |
| 27 | D27 | Downstream | Mainstream | 114.0341667 | 34.93833 |
| 28 | D28 | Downstream | Mainstream | 114.4727778 | 34.87833 |
| 29 | D29 | Downstream | Mainstream | 114.7819444 | 34.97806 |
| 30 | D30 | Downstream | Mainstream | 116.744044 | 36.63918 |
| 31 | D31 | Downstream | Mainstream | 115.4500556 | 35.57639 |
| 32 | D32 | Downstream | Tributary | 116.1277778 | 36.015 |
| 33 | D33 | Downstream | Tributary | 116.8472222 | 35.92528 |
| 34 | D34 | Downstream | Mainstream | 116.2231 | 35.9462 |
| 35 | D35 | Downstream | Mainstream | 116.1976 | 35.9786 |
| 36 | D36 | Downstream | Mainstream | 113.6801 | 34.919 |
| 37 | D37 | Downstream | Mainstream | 116.338 | 36.2975 |
| 38 | D38 | Downstream | Mainstream | 118.3074 | 37.5145 |
| 39 | D39 | Downstream | Mainstream | 118.7218 | 37.8866 |
| 40 | D40 | Downstream | Mainstream | 119.156 | 37.76 |
| 41 | D41 | Downstream | Mainstream | 118.5367959 | 37.60461 |
| 42 | D42 | Downstream | Mainstream | 118.7645148 | 37.7382 |
| 43 | D43 | Downstream | Tributary | 119.1368657 | 37.74957 |
| 44 | D44 | Downstream | Tributary | 119.1304771 | 37.77068 |
| 45 | D45 | Downstream | Tributary | 118.723229 | 38.07323 |
| 46 | D46 | Downstream | Tributary | 118.7239238 | 38.02024 |


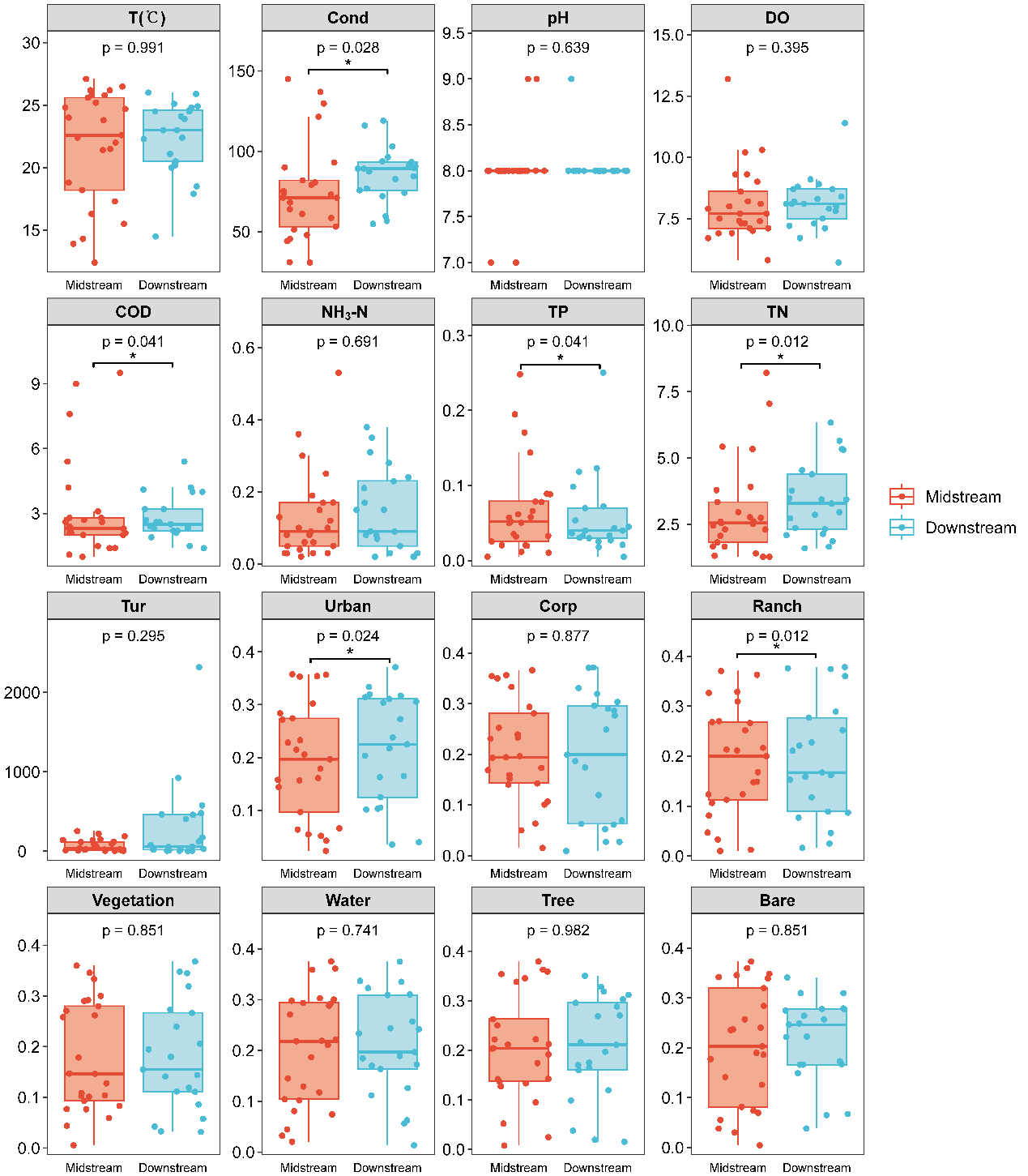


Fig. S1 Box plots of 16 environmental variables. The Kruskal-Wallis test was used to assess differences between different sites, *P < 0.05, ** P < 0.01


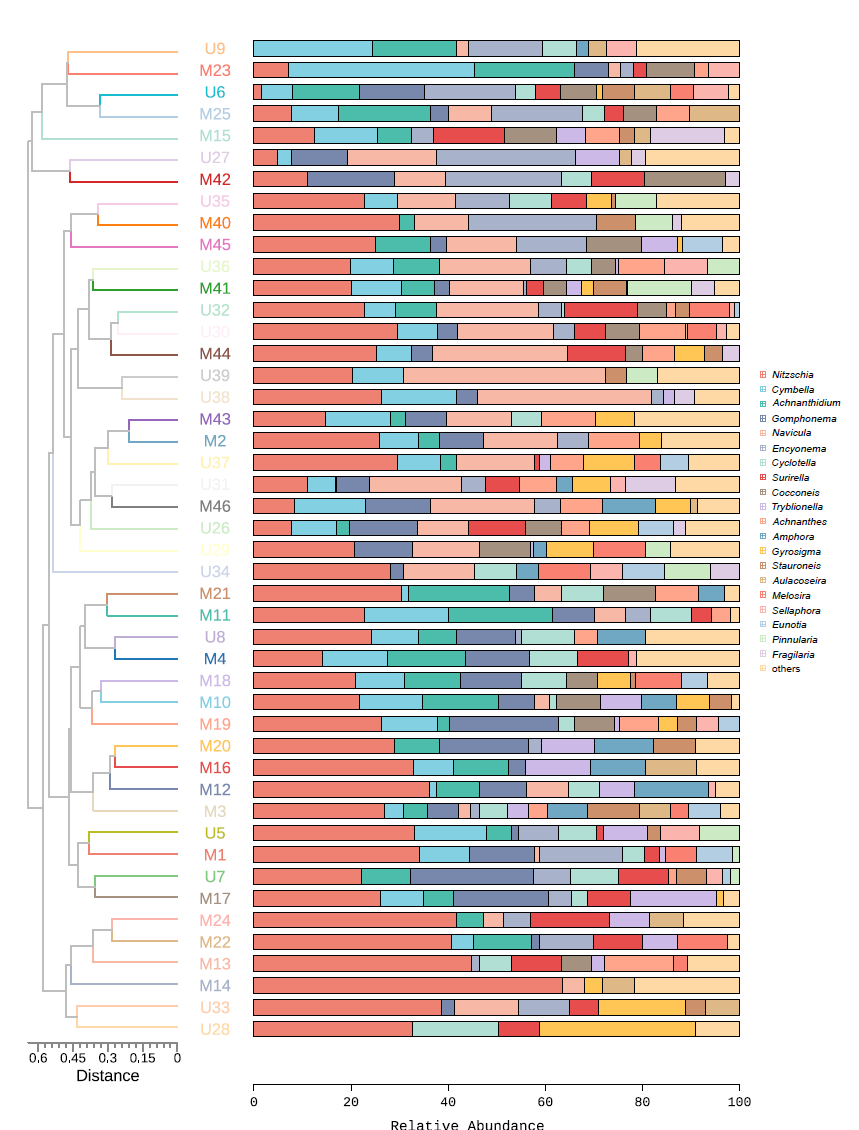


Fig. S2 Relative abundance of benthic diatom genera across sampling sites and clustering dendrogram based on Bray-Curtis distance.


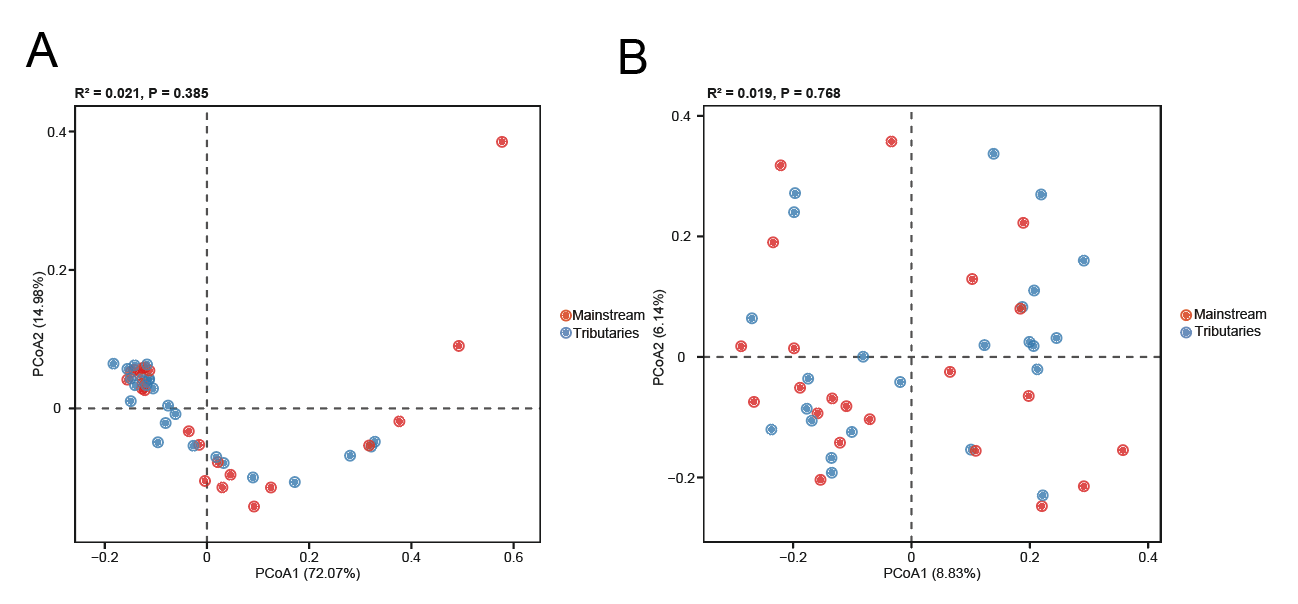


Fig. S3 The PCoA shows the differences in the environment (A) and species (B) between tributaries and the main stream, with no significant differences observed in the ANOVA test.


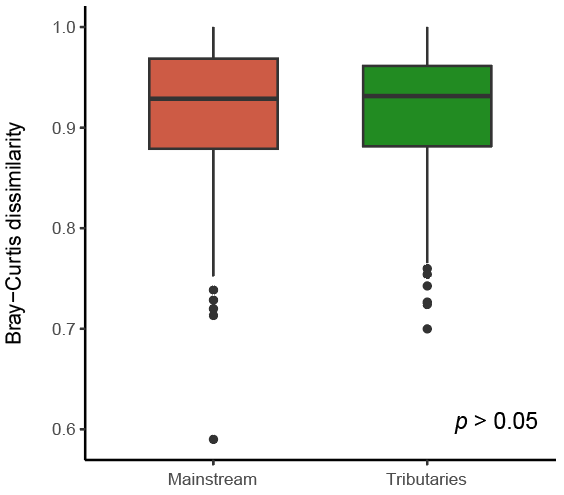


Fig. S4 Boxplot comparing β-diversity (Bray-Curtis dissimilarity) of benthic diatom communities between mainstream and tributaries sites. No significant differences were observed (Wilcoxon rank-sum test, *p* > 0.05).
